# Effects of Age, Hemoglobin Type and Parasite Strain on IgG Recognition of *Plasmodium falciparum*–Infected Erythrocytes in Malian Children

**DOI:** 10.1371/journal.pone.0076734

**Published:** 2013-10-04

**Authors:** Amir E. Zeituni, Kazutoyo Miura, Mahamadou Diakite, Saibou Doumbia, Samuel E. Moretz, Ababacar Diouf, Gregory Tullo, Tatiana M. Lopera-Mesa, Cameron D. Bess, Neida K. Mita-Mendoza, Jennifer M. Anderson, Rick M. Fairhurst, Carole A. Long

**Affiliations:** 1 Laboratory of Malaria and Vector Research, National Institute of Allergy and Infectious Diseases, National Institutes of Health, Rockville, Maryland, United States of America; 2 Faculty of Medicine, Pharmacy and Odontostomatology, University of Bamako, Bamako, Mali; 3 Departamento de Biomedicina Molecular, Centro de Investigación y Estudios Avanzados, México City, México; Walter & Eliza Hall Institute, Australia

## Abstract

**Background:**

Naturally-acquired antibody responses to antigens on the surface of *Plasmodium falciparum*-infected red blood cells (iRBCs) have been implicated in antimalarial immunity. To profile the development of this immunity, we have been studying a cohort of Malian children living in an area with intense seasonal malaria transmission.

**Methodology/Principal Findings:**

We collected plasma from a sub-cohort of 176 Malian children aged 3-11 years, before (May) and after (December) the 2009 transmission season. To measure the effect of hemoglobin (Hb) type on antibody responses, we enrolled age-matched HbAA, HbAS and HbAC children. To quantify antibody recognition of iRBCs, we designed a high-throughput flow cytometry assay to rapidly test numerous plasma samples against multiple parasite strains. We evaluated antibody reactivity of each plasma sample to 3 laboratory-adapted parasite lines (FCR3, D10, PC26) and 4 short-term-cultured parasite isolates (2 Malian and 2 Cambodian). 97% of children recognized ≥1 parasite strain and the proportion of IgG responders increased significantly during the transmission season for most parasite strains. Both strain-specific and strain-transcending IgG responses were detected, and varied by age, Hb type and parasite strain. In addition, the breadth of IgG responses to parasite strains increased with age in HbAA, but not in HbAS or HbAC, children.

**Conclusions/Significance:**

Our assay detects both strain-specific and strain-transcending IgG responses to iRBCs. The magnitude and breadth of these responses varied not only by age, but also by Hb type and parasite strain used. These findings indicate that studies of acquired humoral immunity should account for Hb type and test large numbers of diverse parasite strains.

## Introduction

*Plasmodium falciparum* causes the most severe form of malaria, by some estimates placing more than 3 billion people at risk of disease and killing up to 1 million of them each year [1,2]. The burden of *P. falciparum* malaria is largely carried by the youngest of children living permanently in endemic areas [3]. The development of naturally-acquired immunity to malaria is slow and poorly understood. As children experience multiple *P. falciparum* infections during childhood and adolescence, they develop successive stages of non-sterilizing immunity that protect them from severe and uncomplicated malaria, and eventually suppress their parasite densities [4,5]. This process produces an adult population with asymptomatic parasitemias that are often below the level of microscopic detection in thick blood films. These stages of naturally-acquired immunity are believed to arise in part from repeated exposure to parasite strains expressing different constellations of variant surface antigens (VSAs) on the surface of their host red blood cells (RBCs) [6,7]. The cumulative exposure to VSAs results in a repertoire of parasite strain-specific immune responses that collectively confer some degree of strain-transcending immunity (i.e., premunition) [8].

Considerable evidence suggests that naturally-acquired immune IgG reduces the incidence and severity of malaria syndromes, and limits parasite densities. In 1961, Cohen et al. demonstrated in the Gambia that the passive transfer of gamma globulin from immune adults to very young children with malaria alleviated their illness and reduced their parasite densities [9]. A subsequent study showed that passive transfer of pooled immune IgG from adults living in different malaria-endemic regions of Africa to non/semi-immune Thai patients with drug-resistant malaria was associated with efficient reduction in fever and parasitemia [10]. The antigen specificities and effector mechanisms of passively-transferred immune IgG have not been fully defined. IgG responses to merozoite antigens (e.g., AMA-1, EBA-175, MSP-1, MSP-2) and *P. falciparum* erythrocyte membrane protein 1 (PfEMP1) variants – which constitute a family of adhesins – have all been implicated in protective immunity. Possible mechanisms include neutralizing merozoite invasion of RBCs and opsonizing parasite-infected RBCs (iRBCs). IgG opsonization of iRBCs may weaken the binding of iRBCs to the microvascular endothelium, fix complement to iRBC surfaces, and enhance FcγR- and complement receptor-mediated phagocytosis of iRBCs by blood monocytes and splenic macrophages.

In sub-Saharan Africa, common RBC polymorphisms [sickle hemoglobin (Hb) S, HbC, α-thalassemia, glucose-6-phosphate dehydrogenase (G6PD) deficiency, type O blood group antigen] have been variously associated with protection against *P. falciparum* malaria [11-16], and thus represent human evolutionary adaptations to the morbidity and fatal complications of this disease [17]. A recent meta-analysis, for example, found that HbS heterozygosity (HbAS) and HbC homozygosity (HbCC) significantly reduce the risk of severe malaria >90% compared to HbA homozygosity (HbAA) [18]. It has been proposed that these and related Hb traits (e.g., HbAC) confer malaria protection via innate mechanisms, acquired immune responses, or both. Abnormal display of PfEMP1, the parasite’s major cytoadherence ligand and VSA, on the surface of HbAS, HbAC and HbCC RBCs has been implicated in malaria protection [19]. Specifically, this phenotype has been associated with the weakening of iRBC cytoadherence to microvascular endothelial cells and rosette formation with uninfected RBCs [20-22]. Other innate mechanisms of malaria protection have been proposed for HbAS such as enhanced sickling of iRBCs and oxidation-induced damage to iRBC membranes (with negative consequences for parasite survival) [23,24]. An additional proposed mechanism suggests increased HbAS RBC hemolysis, results in high levels of heme oxygenase-1 (HO-1) in the blood which affects levels of CO; increased HO-1 has also been associated with the production of dysfunctional neutrophils and diminished pro-inflammatory activity [25-29]. Immune-mediated mechanisms have also been proposed for HbAS, including the enhancement of antibody responses to PfEMP1 and perhaps other VSAs (e.g., stevors, rifins) [30,31].

Historically, investigators have relied on serum agglutination assays to measure antibody reactivity to VSAs on intact iRBCs [6,32,33]. For example, Marsh and Howard reported that Gambian children showed *P. falciparum* strain-specific agglutination responses, while Gambian adults showed cross-reactive responses [6]– presumably antibodies directed to a conserved antigen, or a variety of antibodies against many antigens expressed in different strains. Also working with Gambian sera, Newbold et al. confirmed that the agglutinating antibody response is highly parasite strain-specific [34]. More recently, results from agglutination assays were used to categorize *P. falciparum* VSAs into 2 groups: one containing functional VSAs that mediate efficient cytoadherence and another containing a diverse antigen repertoire that evades the immune system [35]. The results of such agglutination assays have correlated with malaria risk (at the time of sample collection) [36], strain specific protection [37,38], and clinical protection in other studies [30,39]. Given these findings, it is clear that the antigenic targets, immune effector functions, and malaria-protective roles of agglutinating IgG responses to intact iRBCs have yet to be fully elucidated.

Studies using Indirect Fluorescent Assays (IFAs) have also concluded that higher IgG titers to iRBCs are associated with reduced fever and parasite burden [40-42]. However, these findings differed from results obtained in passive transfer studies using the *Saimiri sciureus* (squirrel monkey) model, in which antibody titers (measured by IFA) to *P. falciparum* schizont-iRBCs did not correlate with protection (measured by evaluating the course of parasitemia following intravenous inoculation with 50 x 10^6^ iRBCs) [43-45]. One interpretation of this finding is that anti-*Plasmodium* antibodies exert their protective effects at a relatively low concentration and that increasing this concentration by passive transfer of more antibodies does not enhance protection. As technology progressed, investigators used less subjective and more quantitative tests such as ELISA using schizont lysates [46,47] and flow cytometry using intact trophozoite- and schizont-iRBCs to evaluate an individual’s antibody responses to *P. falciparum* laboratory-adapted clones and clinical isolates [39,48-51]. Staalsoe et al., for example, reported a strong correlation between serum agglutination responses to iRBCs and IgG reactivity to iRBCs as measured by flow cytometry [48].

Given that antibodies play such a fundamental role in immunity to *P. falciparum* malaria, we sought to develop a standardized flow cytometry assay to quantify an individual’s antibody reactivity to the surface of iRBCs. To establish and apply this assay, we compared the IgG responses of 176 Malian children aged 3-11 years to a panel of iRBCs: 3 laboratory-adapted parasite lines and 4 short-term-adapted parasite isolates from Mali or Cambodia. We also explored whether IgG responses to these 7 parasite strains increased during a transmission season and whether they differed between age-matched groups of HbAA, HbAS and HbAC children.

## Materials and Methods

### Ethics statement

The Ethics Committee of the Faculty of Medicine, Pharmacy, and Odontostomatology at the University of Bamako, and the Institutional Review Board of the National Institute of Allergy and Infectious Diseases approved this study. Written informed consent was obtained from Malian adults or the parents or guardians of children if they could read French; if they could not, oral consent was obtained and documented by both the adult being consented and a third party witness. This study is registered with Clinicaltrials.gov, number NCT00669084.

### Study design

The present study is part of a larger 4-year study of malaria incidence conducted in 3 neighboring rural villages (Kenieroba, Bozokin, Fourda) located in the Koulikoro region of Mali, which experiences intense seasonal transmission of *P. falciparum* between June and December. Further details of this study site and the overall cohort of 1586 children have been previously described [52]. All children were typed for Hb type, α-thalassemia, G6PD deficiency, and ABO/Rh blood group antigens, as described [52]. Parents or guardians were actively encouraged to bring their children to our study clinic whenever they developed fever or other symptoms of malaria.

Within the cohort of 1586 children, we established a sub-cohort of 200 children aged 3-11 years from Kenieroba and Fourda in May 2009. Each HbAS (n=73) and HbAC (n=27) child was matched to a HbAA child (n=100) of the same age. Whenever possible, each child pair was also matched for sex, ABO/Rh blood group antigens, and G6PD and α-thalassemia genotypes. Before (May) and after (December) the 2009 transmission season, 5-8 ml of venous blood were collected into sodium heparin-containing Vacutainer tubes (BD Biosciences, Franklin Lakes, NJ). Whole blood was processed using Ficoll-Paque PLUS (GE Healthcare, Niskayuna, NY); plasma was separated by centrifugation and stored at -80°C until use. After following these children for malaria episodes during the entire transmission season, we obtained and processed venous blood from all available children in December 2009. Uncomplicated malaria was defined as axillary temperature >37.5°C (or history of fever in the previous 48 hours) with or without additional symptoms (e.g., headache, body aches, malaise), plus *P. falciparum* parasitemia (any density) on thick blood smear, and no other etiology of febrile illness (e.g., respiratory tract infection) discernible on clinical examination. Children were treated for uncomplicated malaria with artesunate and amodiaquine, as described [52].

For the current study, out of the 200 children, we only analyzed data from 176 Malian children from whom both May (pre) and December (post) samples were available. The 176 children included 89 HbAA, 61 HbAS and 26 HbAC children. Out of the 176 children, 6 (HbAA), 4 (HbAS) and 2 (HbAC) were unmatched for age.

Sera from malaria-naïve American adults and RBCs for *in-vitro* parasite culture were obtained from Interstate Blood Bank (Memphis, TN).

### Laboratory-adapted *P. falciparum* lines and short-term-adapted *P. falciparum* isolates

*P. falciparum* lines (FCR3, D10, PC26) were cultured in O+ RBCs at 2% hematocrit in complete media (CM) consisting of RPMI 1640 w/L-Glutamine + 25 mM HEPES + 50 mg/l hypoxanthine (KD Medical, Columbia, MD), supplemented with 30 ml 7.5% sodium bicarbonate (Gibco, Life Technologies, Grand Island, NY), 1 ml 10 µg/ml gentamicin (Gibco, Life Technologies) and 0.5% Albumax II (Invitrogen, Life Technologies). Cultures were maintained at 37°C in a 25-cm^2^ flask (Corning, Tewksbury, MA) at 37°C in an atmosphere of 5% O_2_, 5% CO_2_ and 90% N_2_. FCR3 was reported as originating from an individual living in Fajara, The Gambia, in 1976 [53]. D10 was cloned from the *P. falciparum* isolate FC27 originating from Madang Province, Papua New Guinea [54,55]. PC26 is described as originating from Peru [56]. *P. falciparum* isolates were obtained from Malian children (KN1254, KN1068) [52] or Cambodian adults (CP803, CP806) [57] with uncomplicated malaria, and adapted to *in-vitro* culture in CM modified to contain 1% Albumax II. To minimize the confounding effects of antigenic variation in antibody reactivity assays, *P. falciparum* lines and isolates were grown for 1 week to high parasitemia and then aliquoted and cryopreserved. Before each assay, cryopreserved parasites were thawed and grown until they matured to late trophozoites/early schizonts. All parasite lines and isolates were confirmed to be negative for *Mycoplasma* contamination using the Mycotrace PCR Detection Kit (PAA Laboratories, Morningside, Australia).

### Multicolor flow cytometry detection of antibody reactivity to intact iRBCs

Flow cytometry detection of antibody reactivity to intact iRBCs was adapted from various protocols [39,48,58-60] and has been previously described [52]. Synchronized parasite cultures (<1% parasitemia) were washed in PBS, pH 7.2, supplemented with 2% heat-inactivated fetal bovine serum (Gibco, Life Technologies) (FPBS), and diluted to 1% hematocrit. A 200-µl suspension of washed iRBCs was added to a single well of a 96-well plate (Corning Costar, Corning, NY) and centrifuged at 800xg for 5 min. The supernatant was gently removed and replaced with 200 µl of heat-inactivated plasma (diluted 1:20 in FPBS) collected from Malian children, Malian adults, or heat-inactivated sera from malaria-naïve American adults (negative). iRBC-plasma mixtures were constantly agitated at 500 rpm using a Heidolph Titramax 100 platform shaker (Heidolph, Schwabach, Germany) for 30 min at room temperature. iRBCs were pelleted, washed once with FPBS, and resuspended in freshly prepared “Mix A” [0.5 µl/ml Syto61 (Invitrogen, Life Technologies), 3 µl/ml Alexa Fluor 488-conjugated goat anti-human IgG (H+L) (Invitrogen, Life Technologies), and 3 µl/ml PE-conjugated mouse anti-human IgM (µ chain) (BD Pharmingen, San Diego, CA)], as well as single reagent controls where appropriate. iRBCs suspensions were constantly agitated at 500 rpm for 30 min at room temperature, washed once and resuspended in FPBS. We gated on singlet cell populations (FSC-H vs FSC-A) (>75% of total), excluded doublets/aggregates and debris (data not shown). ≥5000 Syto61-positive events were acquired from each well and analyzed using an Accuri C6 flow cytometer and the Accuri CFlow Plus software (BD Biosciences, San Jose, CA). The gating strategy is shown in Figure **S1**.

### ELISA

The standardized ELISA was previously described [61]. The absorbance of each test sample was converted into ELISA units using a standard curve generated by serially diluting the standard in the same plate. Apical membrane antigen 1 (AMA-1) [62] and the 42-kDa fragment of merozoite surface protein 1 (MSP-1) [63] were kindly provided by Dr. David Narum (NIAID). Erythrocyte-binding antigen 175 KDa (EBA-175) region II [64] was kindly provided by Dr. Annie Mo (DMID/NIAID). Merozoite surface protein 2 (MSP-2) from the 3D7 *P. falciparum* line [65] was kindly provided by Dr. Robin Anders (La Trobe University, Melbourne, Australia). The minimal antibody detection level was 44 ELISA units; all responses below this level were assigned a value of 22 ELISA units.

### Statistical analysis

Multiple nonparametric comparisons were made using a Kruskal-Wallis test; if significant differences were found, we used Dunn’s multiple comparison test in follow-up analyses. For each plasma sample, the proportion of iRBCs recognized by IgG (“% recognition”) was calculated. For each parasite strain, we determined a threshold value (mean + 2 standard deviations) for % recognition using data from 4 malaria-naïve American adult plasma samples tested in 5 independent assays (per parasite evaluated). A child with a % recognition value greater than the threshold value was defined as a responder. The proportion of responders in the group of children tested was defined as “% responders.” Fisher’s exact test was used to compare the changes in % responders between pre- and post-transmission season samples. Qualitative data from multiple groups were analyzed using Chi-square likelihood ratio test; if significant differences were found, we calculated odds ratios (ORs) to determine which group was significantly different from the others. Nominal logistic fit was used to analyze data indicating whether or not malaria was experienced, and Proportional hazards fit was used to analyze data indicating the time to first malaria episode. For contingency analysis, Chi-square likelihood ratio test was used to compare 2 parasite strains; p-values were then Holm’s corrected and provided. For each parasite strain, % recognition values for pre- and post-transmission season samples were compared using Wilcoxon matched-pairs signed rank test. Statistical analyses were performed using GraphPad Prism 5 (GraphPad Software, La Jolla, CA) and JMP (SAS, Cary, NC) statistical software. p-values <0.05 were deemed significant.

## Results

### Increasing age and HbAS are associated with reduced malaria risk in Malian children

We analyzed data from 176 Malian children aged 3-11 years who provided a plasma sample before and after the 2009 malaria transmission season. Of the 89 HbAA, 61 HbAS and 26 HbAC children, only 6 (HbAA), 4 (HbAS) and 2 (HbAC) were unmatched for age due to missing samples. Only 3 children (aged 7, 8 and 10 years) had asymptomatic *P. falciparum* parasitemia in a thick blood smear at enrollment. No child had detectable parasitemia at the end of the transmission season. The 176 children experienced a mean of 1.23 malaria episodes during the 7-month transmission season. Malaria incidence rates stratified by Hb type and age are shown in Table **1**. HbAS, but not HbAC, children had significantly fewer malaria episodes than HbAA children (0.75 *vs.* 1.47, p=0.0004 for HbAS; 1.57 *vs.* 1.47, p=0.6769 for HbAC; Kruskal-Wallis test followed by Dunn’s multiple comparison test). Children aged 9-11 years experienced significantly fewer malaria episodes than children aged 6-8 years (0.7 *vs.* 1.26, p=0.0458) or 3-5 years (0.7 *vs.* 1.66, p<0.0001).

**Table 1:** Malaria incidence rates stratified by Hb type and age.

**Hb type**	**3-5 years**		**6-8 years**		**9-11 years**		**Total**
	n	(Cases)^a^	n	(Cases)	n	(Cases)	n (Cases)
AA	28	(2.10)	35	(1.40)	26	(0.88)	89 (1.47)
AS	23	(0.95)	20	(0.80)	18	(0.44)	61 (0.75)
AC	8	(2.12)	12	(1.66)	6	(0.66)	26 (1.57)
Total	59	(1.66)	67	(1.26)	50	(0.70)	176 (1.23)

^a^ Arithmetic mean number of malaria episodes during the 7-month transmission season.

In a multivariate regression analysis accounting for age and Hb type, we found that the odds of experiencing ≥1 malaria episode decreased significantly with age (Table **2**). HbAS, but not HbAC, children were also less likely to develop a malaria episode than HbAA children (OR=0.40, 95% CI 0.19-0.82, p=0.012) (Table **2**). The time to first malaria episode during the 2009 transmission season increased significantly with age and was longer in HbAS than in HbAA children (RR=0.47, 95% CI 0.30-0.71, p<0.001) (Table **2**). In another multivariate regression analysis accounting for age and Hb type, we found that IgG responses to multiple parasite strains were not associated with protection from malaria or delayed onset of malaria (data not shown).

**Table 2:** Multivariate regression analysis: effects of age and Hb type on malaria risk.^a^

	Whether malaria was experienced				Time to first malaria episode		
	OR^b^		(95% CI)	p-value	RR^c^	(95% CI)	p-value
6-8 vs. 3-5 y		0.36	(0.14-0.85)	0.019	0.56	(0.39-0.90)	0.014
9-11 vs. 6-8 y		0.42	(0.19-0.92)	0.030	0.61	(0.36-0.99)	0.047
9-11 vs. 3-5 y		0.15	(0.06-0.37)	<0.001	0.36	(0.21-0.59)	<0.001
AS vs. AA		0.40	(0.19-0.82)	0.012	0.47	(0.30-0.71)	<0.001
AC vs. AA		1.25	(0.45-3.89)	0.673	1.07	(0.63-1.74)	0.777

^a^ Age and Hb type were analyzed for their effect on 2 measures of malaria risk: whether or not malaria was experienced (in the cohort of 176 children), and the time to first malaria episode (in the subset of 117 children who experienced ≥1 malaria episode).

^b^ Odds ratio.

^c^ Relative risk.

### Optimization of assay to measure antibody recognition of the surface of iRBCs

In optimizing the flow cytometry-based assay, we found that antibody-mediated agglutination of iRBCs was minimized in samples with parasitemia <1% and hematocrit <1%. Under these conditions, 1:10 and 1:20 dilutions of plasma produced better signal-to-noise ratios than 1:5 and 1:40 dilutions of plasma (data not shown). To use samples sparingly, all subsequent experiments were performed with 1:20 dilutions of plasma. In assays using sera from 4 malaria-naïve American adults, there was minimal assay-to-assay variation (measured by standard deviation) in the proportion of iRBCs recognized by IgG (“% recognition”) (Figure **S2**). Greater assay-to-assay variation in % recognition values (Figure **S2**) and mean fluorescence intensities (MFIs) of iRBCs (data not shown) were observed for Malian compared to American adult samples. We therefore transformed the data by defining Malian children as IgG “responders” if they showed a % recognition value greater than the mean + 2 standard deviations of the % recognition value of American adults. The binary readouts “responder” and “non-responder” were then analyzed instead of % recognition values or MFIs. While each child’s IgG responded to at least 1 *P. falciparum* strain at one or both time points, no child’s IgM responded to any of the 7 parasite strains (data not shown).

### The proportion of responders to diverse *P. falciparum* strains increases during the transmission season

**Figure 1:**
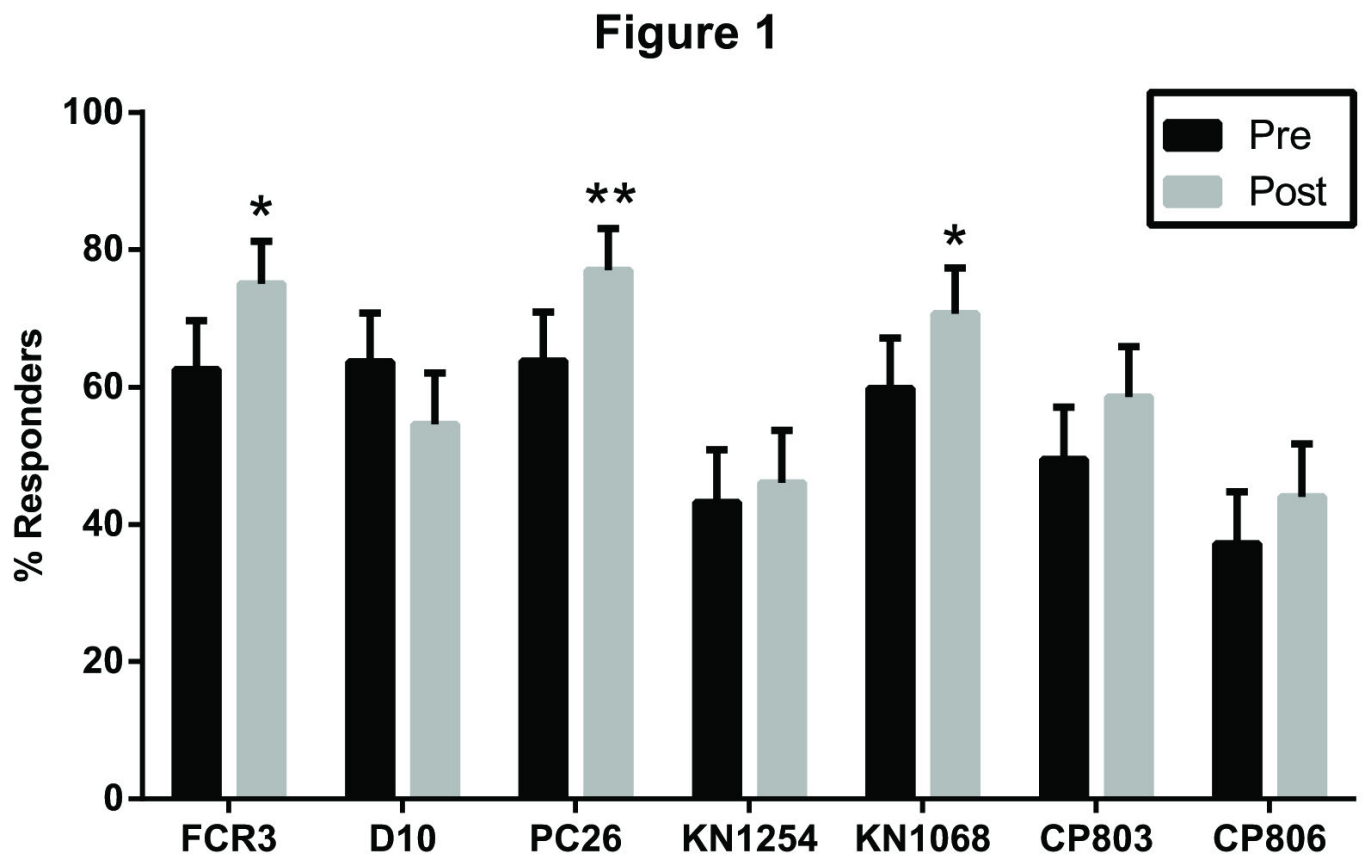
Comparison of % responders before and after the 2009 transmission season. The proportion of iRBCs recognized by IgG (% recognition) was measured by flow cytometry before and after the 2009 transmission season in all 176 children. A child with a % recognition value greater than the mean + 2 standard deviations of the % recognition value in American adults was defined as a “responder.” The % responders to each parasite strain before and after the transmission season was compared using Fisher’s exact test. *p<0.05, **p<0.001. Error bars shown are the upper 95% Confidence Interval for the % responder estimates.

Before the transmission season, 97.2% (171/176) of the children recognized ≥1 of 7 parasite strains. The frequency of responders ranged from 37% to 77% depending on the parasite strain tested (Figure **1**). For 3 parasite strains, the % responders increased significantly during the transmission season (FCR3, p=0.0155; PC26, p=0.0096; KN1068, p=0.0425; Fisher’s exact test). To determine whether IgG recognition of iRBCs also increased, we calculated the difference between % recognition values in each child’s paired plasma samples (assayed simultaneously). Over the transmission season, % recognition values increased significantly for 5 parasite strains (FCR3, p=0.0006; PC26, p<0.0001; KN1068, p=0.0025; CP803, p=0.0003; CP806, p=0.0005; Wilcoxon matched-pairs signed rank test) (Figure **S3**). In contrast, % recognition values for D10 decreased (p<0.0001) and those for KN1254 did not change (Figure **S3**). Taken together, these data indicate that IgGs from Malian children generally recognize diverse parasite strains and that this IgG recognition increases over a single transmission season.

### Age and Hb type correlations with IgG recognition of iRBCs

**Figure 2:**
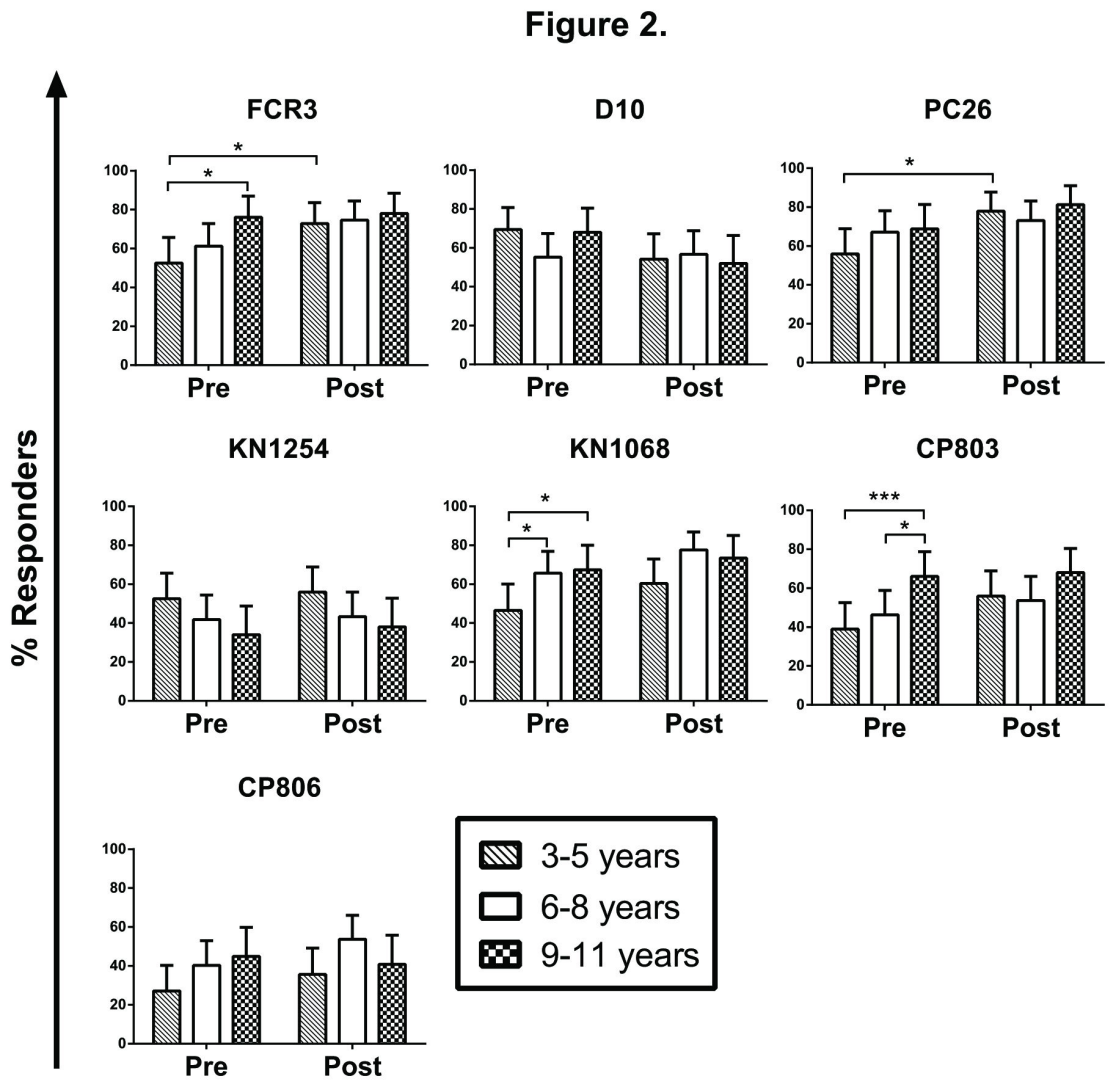
Effect of age on IgG recognition of different parasite strains. The proportions of children responding to each parasite strain were compared between age groups, before and after the transmission season. The age effect at each time point was evaluated using Chi-square likelihood ratio test; significant findings were then confirmed by Odds ratio test. The seasonal effect in each age group was evaluated using Fisher’s exact test. *p<0.05, **p<0.01, ***p<0.001. Error bars shown are the upper 95% Confidence Interval for the % responder estimates.

To evaluate the relationship between age and IgG recognition of parasite strains, we stratified children *a priori* into 3 age groups: 3-5, 6-8 and 9-11 years. Before the transmission season, the % responders to 3 parasite strains (FCR3, KN1068 and CP803) were significantly greater in children 9-11 years old than in those 3-5 years old (76% *vs.* 47% for FCR3, p<0.05; 67% *vs.* 47% for KN1068, p<0.05; 66% *vs.* 39% for CP803, p<0.001; Chi-square likelihood ratio test) (Figure **2**). The % responders to D10, PC26, KN1254 and CP806 did not differ by age. After the transmission season, no age-associated differences in % responders to any of the 7 parasite strains were seen (Figure **2**). We also evaluated the seasonal effect on % responders by comparing pre- and post-transmission season data within each age group. The only significant changes were increases in % responders to FCR3 (p=0.0357, Fisher’s exact test) and PC26 (p=0.0183) in children aged 3-5 years (Figure **2**).

**Figure 3:**
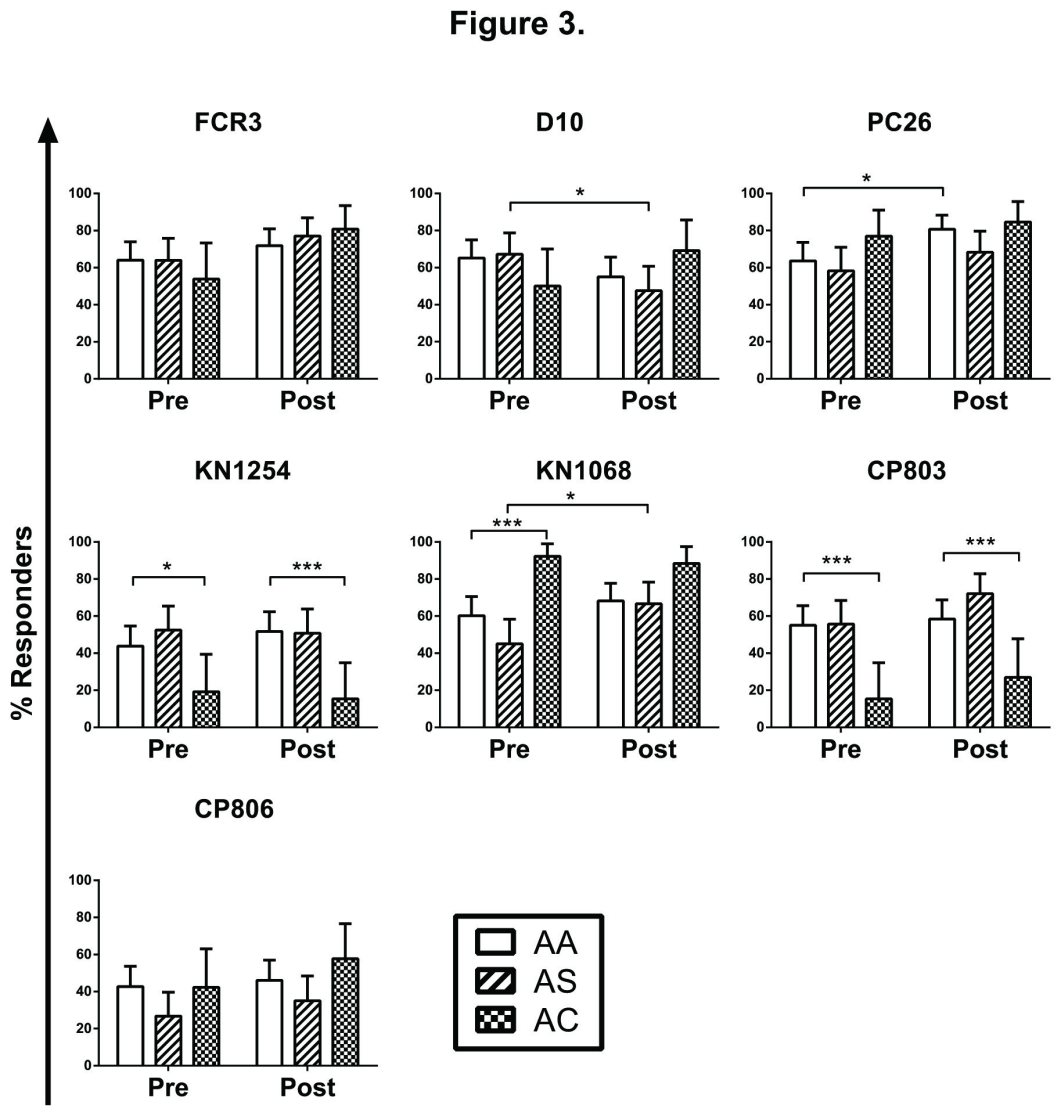
Effect of Hb type on IgG recognition of different parasite strains. The proportions of children responding to each parasite strain were compared between Hb types, before and after the transmission season. The age effect at each time point was evaluated using Chi-square likelihood ratio test; significant findings were then confirmed by Odds ratio test. The seasonal effect of each Hb type was evaluated using Fisher’s exact test. *p<0.05, **p<0.01, ***p<0.001. Error bars shown are the upper 95% Confidence Interval for the % responder estimates.

We next evaluated the relationship between Hb type and IgG recognition of parasite strains. Before the transmission season, the % responders were significantly different between HbAA and HbAC children for 3 of 7 parasite strains (44% *vs.* 19% for KN1254, p=0.0185; 60% *vs.* 92% for KN1068, p=0.0008; 55% *vs.* 15% for CP803, p=0.0002; Chi-squared likelihood ratio test followed by an Odds Ratio test) (Figure **3**). After the transmission season, the % responders were significantly different between HbAA and HbAC children only for these same 3 parasite strains (52% *vs.* 15% for KN1254, p=0.0006; 68% *vs.* 88% for KN1068, p=0.0295; 58% *vs.* 27% for CP803, p=0.0042) (Figure **3**). No such differences were observed between HbAA and HbAS children for any of the 7 parasite strains, either before or after the transmission season. We also evaluated whether Hb type was associated with changes in % responders during the transmission season. HbAA children showed increased % responders to PC26 (64% to 81%, p=0.018, Fisher’s exact test), while HbAS children showed increased % responders to KN1068 (45% to 67%, p=0.027) and decreased % responders to D10 (67% to 48%, p=0.0436) (Figure **3**). No other significant changes in % responders were found. These data indicate that IgG recognition of iRBCs can vary significantly by host factors (i.e., age and Hb type), seasonality and parasite strain.

### The breadth of IgG responses to parasite strains increases with age in HbAA, but not in HbAS or HbAC, children

**Figure 4:**
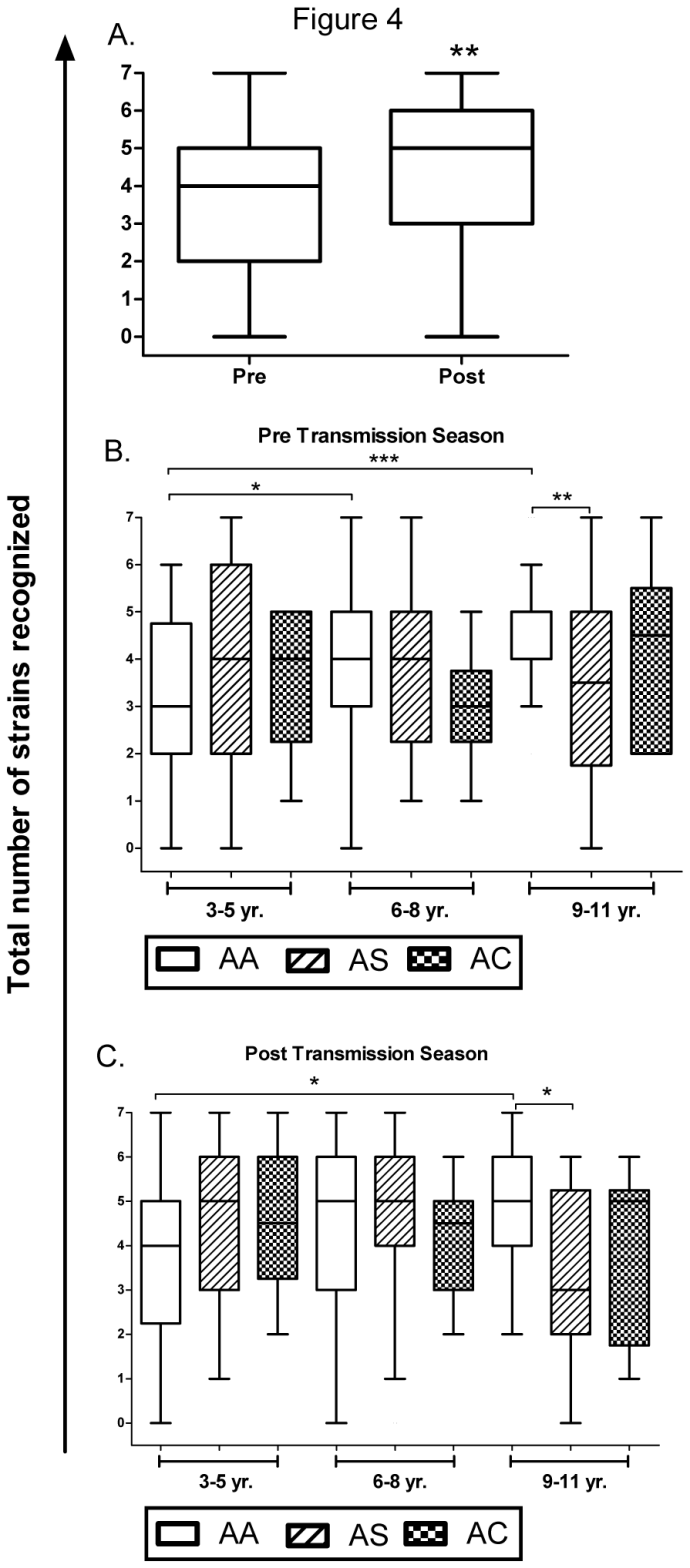
Breadth of IgG responses to parasite strains stratified by season, Hb type and age. The number of parasite strains (range, 0-7) recognized by IgG were counted and stratified by season, Hb type and age. **a**, Tukey whisker plots showing the total number of parasite strains recognized by IgG in paired plasma samples taken before and after the 2009 transmission season. p-values were calculated using Wilcoxon matched-pairs signed rank test. **p<0.01. **b**, **c**, Tukey whisker plots showing the total number of parasite strains recognized by IgG before (**b**) and after (**c**) the 2009 transmission season, stratified by age and Hb type. p-values were calculated using Kruskal-Wallis test followed by Dunn’s multiple comparison test. *p<0.05, **p<0.01, ***p<0.001.

We measured pair-wise correlations between the IgG responses to all 7 parasite strains. Before the transmission season, an IgG response to 1 parasite strain significantly correlated with that to another parasite strain in 38% (8/21) of combinations tested (Table **3**). All significant correlations were positive correlations (i.e., children with a positive response to one parasite strain had a positive response to another strain). After the transmission season, this proportion increased to 62% (13/21) (Table **4**). Similarly, all significant correlations were positive ones. To evaluate the breadth of IgG responses, we counted the number of parasite strains recognized by each child’s IgG before and after the transmission season. As expected, the median number of parasite strains recognized by the entire cohort of children increased significantly (4 to 5, p=0.0013, Wilcoxon matched-pairs signed rank test) during the transmission season (Figure **4a**). To evaluate the effect of host factors on the breadth of IgG responses, we stratified the number of parasite strains recognized by age and Hb type. Before and after the transmission season, the number of parasite strains recognized by IgG increased significantly with age only in HbAA children (Figures **4b, c**). The only other significant differences were that 9- to 11-year-old HbAA children recognized more parasite strains than their HbAS counterparts before (median number recognized 5 *vs.* 3.5, p=0.0094, Mann-Whitney test) and after (5 *vs.* 3, p=0.0208) the transmission season (Figures **4b, c**).

**Table 3:** Contingency analysis to determine whether IgG responses to parasite strains correlate with each other: pre-transmission season.^a^

	**D10**	**PC26**	**KN1254**	**KN1068**	**CP803**	**CP806**
**FCR3**	0.286	0.053	0.003	1	0.002	0.008
**D10**		1	1	1	0.613	0.613
**PC26**			0.613	0.002	0.027	0.002
**KN1254**				0.286	0.002	0.293
**KN1068**					1	0.018
**CP803**						0.286

^a^ Chi-square likelihood ratio test was used to compare IgG responses to two parasite strains. Holm’s corrected p-values are shown.

**Table 4:** Contingency analysis to determine whether IgG responses to parasite strains correlate with each other: post-transmission season.^a^

	**D10**	**PC26**	**KN1254**	**KN1068**	**CP803**	**CP806**
**FCR3**	0.253	0.002	0.002	0.006	0.002	0.013
**D10**		0.013	0.794	0.002	0.794	0.794
**PC26**			0.463	0.002	0.054	0.012
**KN1254**				0.794	0.002	0.006
**KN1068**					0.024	0.002
**CP803**						0.346

^a^ Chi-square likelihood ratio test was used to compare IgG responses to two parasite strains. Holm’s corrected p-values are shown.

### IgG responses to 2 parasite strains correlate with subsequent malaria risk

Next we investigated whether an IgG response to particular parasite strains before the transmission season correlated with subsequent malaria risk (Table **5**). After adjusting for age and Hb type, only an IgG response to the Cambodian parasite isolate CP803 correlated with protection against malaria (OR= 0.28, 95% CI 0.10-0.75, p=0.011); however, this IgG response did not correlate with a longer time to first malaria episode (Table **5**). Surprisingly, an IgG response to the Malian parasite isolate KN1254 correlated not only with increased risk of malaria (OR=4.40, 95% CI 1.69-12.5, p=0.002) but also a shorter time to first malaria episode (RR=1.78, 95% CI 1.15-2.74, p=0.008) (Table **5**).

**Table 5:** Multivariate regression analysis: effects of parasite strain-specific IgG responses on malaria risk.^a^

	Whether malaria was experienced				Time to first malaria episode		
	OR^b^	(95% CI)	p-value		RR^c^	(95% CI)	p-value
FCR3	1.04	(0.43-2.54)	0.925		0.95	(0.61-1.48)	0.832
D10	1.22	(0.55-2.66)	0.616		1.07	(0.72-1.61)	0.717
PC26	0.54	(0.21-1.29)	0.170		0.80	(0.53-1.23)	0.315
KN1254	4.40	(1.69-12.5)	0.002		1.78	(1.15-2.74)	0.008
KN1068	0.76	(0.31-1.82)	0.549		0.82	(0.53-1.25)	0.355
CP803	0.28	(0.10-0.75)	0.011		0.71	(0.43-1.15)	0.165
CP806	0.81	(0.35-1.87)	0.622		0.82	(0.53-1.25)	0.375

^a^ Adjusted for age and Hb type.

^b^ Odds ratio.

^c^ Relative risk.

### The breadth of IgG responses to parasite strains correlates with IgG titers to merozoite antigens before, but not after, the transmission season

**Figure 5:**
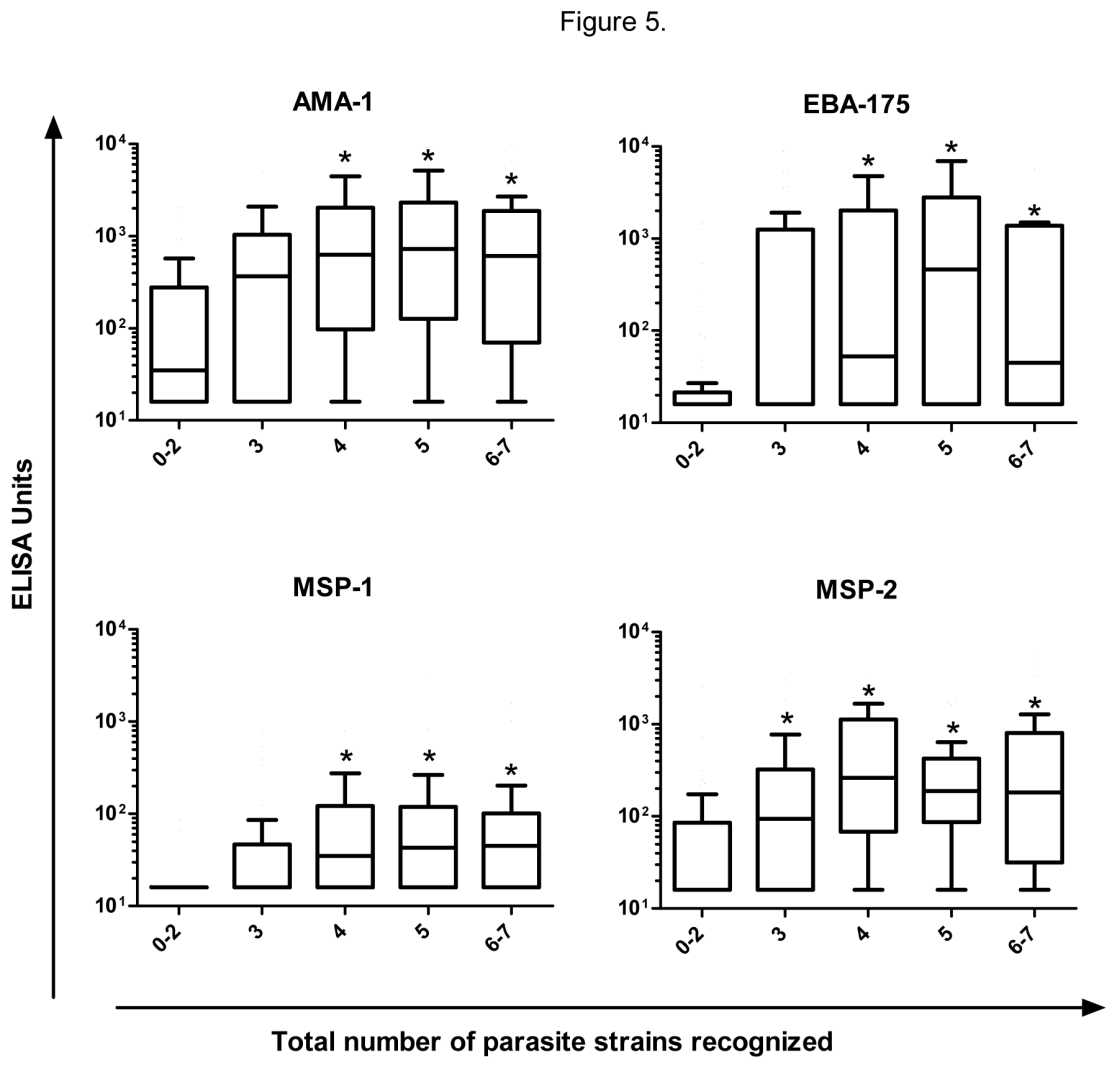
The breadth of IgG responses to parasite strains correlates with increased IgG titers to merozoite antigens. IgG titers to 4 merozoite antigens (AMA-1, EBA-175, MSP-1, MSP-2) were measured in plasma obtained before the transmission season, and stratified by the total number of parasite strains recognized by IgG. Tukey whisker plots are shown. IgG titers were compared using Kruskal-Wallis test followed by Dunn’s multiple comparison test. *p<0.05. The numbers of children recognizing 0-2, 3, 4, 5 and 6-7 parasite strains were 45, 34, 32, 36 and 30.

Finally, we measured correlations between the total number of parasite strains recognized by IgG and the IgG titers to 4 recombinant merozoite antigens (AMA-1, EBA-175, MSP-1, MSP-2). Before the transmission season, IgG titers to all 4 antigens were significantly (p<0.05) higher in groups of children recognizing 4, 5 or 6-7 parasite strains than in the group of children recognizing 0-2 parasite strains (Figure **5**). IgG titers to MSP-2 were also significantly (p<0.05) higher in the group of children recognizing 3 parasite strains than in the group of children recognizing 0-2 parasite strains (Figure **5**). After the transmission season, none of these correlations remained significant.

## Discussion

Antibodies to iRBC surface antigens are a potentially important component of the immune response against erythrocytic-stage malaria parasites. Previous studies have clearly demonstrated that passive transfer of antibodies can alleviate morbidity and reduce parasite densities in patients with malaria [9]. However, it is not known whether these protective antibodies recognize merozoite antigens, iRBC surface antigens, or both. We have developed and optimized a relatively high-throughput, flow cytometry assay to evaluate humoral immune responses to iRBC surface antigens. Using this assay to rapidly test 352 plasma samples against 7 parasite strains, we found that IgG from healthy Malian children recognize genetically-diverse parasite isolates from multiple geographic origins (including Mali). These IgG responses generally increased over a single 7-month transmission season and are influenced by host factors (age and Hb type) and the parasite strain used. Interestingly, the breadth of IgG responses to iRBCs increased with age in HbAA, but not HbAS or HbAC, children.

Our data suggest that our assay identifies both strain-specific and strain-transcending IgG responses to iRBCs. For example, while only 38% of parasite strain combinations tested before the transmission season showed significant pairwise correlations between IgG responses, this number increased to 62% after the transmission season. This finding, and the observation that IgG responses to parasite strains generally increased over the transmission season, suggests that Malian children actively develop strain-transcending IgG responses to iRBC surface antigens. The “strain-transcending” phenotype can be explained by either 1) the child produced antibody which can recognize multiple parasites, or 2) the child produced multiple strain-specific IgGs, or a mixture of both. Further study is required to answer this issue. This seasonal effect on IgG recognition of iRBCs is consistent with results from Giha et al., who reported an increase in serum agglutination of iRBCs over a transmission season in eastern Sudan [66]. Other pairs of IgG responses did not correlate at all, suggesting that each of two IgG repertoires recognize a constellation of parasite strain-specific antigens. These data are consistent with those of other studies showing that sera from children with malaria agglutinated autologous but not heterologous parasite isolates [6,67,68]. While our study demonstrates that Malian children are able to recognize genetically- and geographically-distinct parasites, our data emphasize the importance of evaluating IgG reactivity against many different parasite strains.

Our assay also detected age effects on IgG recognition of iRBCs. Before the transmission season, IgG recognition of iRBCs (FCR3, KN1068, CP803) increased with age. However, this age effect was not observed after the transmission season, suggesting that younger children achieved IgG responses that approximated those of their older counterparts. The age effect observed before the transmission season may therefore be the result of waning IgG responses during the preceding dry season. The lack of a significant seasonal effect in older children is presumably due to plateauing of IgG responses to adult levels. Previous studies in Gambian, Sudanese, Nigerian and Tanzanian adults showed that antibodies can recognize and agglutinate diverse parasite strains from Ghana and Tanzania [6,9,30,69]. Our study corroborates these findings by showing that IgG from older Malian children also recognize diverse parasite strains from Peru, Papua New Guinea, Cambodia, Mali and The Gambia.

The effects of Hb type on IgG recognition of *P. falciparum* antigens have only recently been investigated. Using a recombinant protein microarray, Tan et al. found that HbAS and HbAC were not associated with enhanced *P. falciparum*-specific IgG responses in Malian children [70]. In the same cohort of 176 children studied here, we found that HbAS, but not HbAC, children have significantly lower titers to two recombinant merozoite antigens (EBA175 and MSP2) than their paired HbAA counterparts [71]. While we find no consistent effect of Hb type on IgG recognition of iRBCs in the present study, IgG from HbAC children did recognize 3 of 7 parasite strains significantly differently than IgG from HbAA and HbAS children. Our study thus indicates that the effect of Hb type on iRBC recognition should be interpreted carefully, and should account for age and parasite strain used. Assessing the effects of Hb type on the acquisition of strain-specific and strain-transcending IgG will likely benefit from studying a much larger panel of parasite isolates obtained from the same study population.

As in other studies [12,72-74], we found that increasing age and HbAS are associated with reduced malaria risk in our study population. Previous studies have proposed that the presence of asymptomatic parasitemia at the time of plasma collection correlates with higher protective IgG responses [38,75]. In the present study, however, these confounding effects were not relevant since only 3 children had asymptomatic parasitemia before the transmission season. After adjusting for age and Hb type, IgG recognition of the Cambodian parasite isolate CP803 correlated with protection against malaria. One explanation for this finding is that CP803 incidentally expresses VSAs that are very similar to those expressed by parasites circulating and causing malaria in our cohort of Malian children in 2009. Our finding that IgG recognition of the Malian parasite isolate KN1254 correlated strongly with increased malaria risk is more difficult to explain. The KN1254 parasite isolate was obtained from a 15-year-old HbAA child who presented with severe malaria (as defined by the WHO [76]). While this clinical presentation is rare in this age group, the VSAs expressed by KN1254 (or other very much like them) are almost certainly not rare. This is because, before the transmission season, this Malian parasite isolate was recognized by 43% of children in our study.

Our study has several limitations. First, the Malian and Cambodian parasite isolates we used are not clonal (data not shown). This finding, and the fact that *var* genes switch during *in vitro* culture, makes it difficult to use these parasite isolates as reagents with stable phenotypes in future studies. Second, we have not identified the parasite antigens recognized by the IgG of children in our study. Recently, however, Chan et al. made a very compelling argument in favor of PfEMP1 being the major target of anti-VSA antibodies [7]. Third, the large scale of this study required that we compare flow cytometry data collected over multiple days, which has the potential of introducing assay-to-assay variability. To minimize this variability, we analyzed the binomial readout responders/non-responders instead of % recognition values or MFIs. Fourth, we did not include a positive human IgM control in our assays as it was not available. Therefore, it might be possible that some of the IgG signals we detected by the anti-IgG antibody (which recognizes heavy and light chains) came from cross-reactivity of the anti-IgG antibody to IgM. However, we believe the cross-reactivity to non-specific IgM was minor, as all 4 malaria naïve American adult plasma samples tested in 5 independent assays always showed <6% responses with the anti-IgG antibody. We did not detect IgM with any of the samples in the current study: however, when evaluating autologous plasma and parasites we were able to detect IgM in 1 of the 48 children tested, suggesting that IgM responses were not very robust in this cohort [52]. Moreover, the rarity of IgM antibodies that react against the surface of iRBCs has also been known and documented since the 1980’s [77]. Despite these limitations, we believe this semi-quantitative assay can rapidly measure immune IgG responses to iRBC surface antigens. For example, this assay has been successfully implemented in Mali to investigate how IgG responses influence parasite clearance rates in response to artesunate [52]. Since this assay rapidly tests numerous plasma samples against multiple parasite strains, it may also be useful in evaluating the breadth and magnitude of naturally-acquired IgG responses to iRBC surface antigens and their relationship to malaria protection.

## Supporting Information

Figure S1 **Gating strategy to assess IgG and IgM recognition of parasite-infected red blood cells (iRBCs).** Representative gating strategy for iRBCs recognized by IgG or IgM. **a**, The population of RBCs and iRBCs was gated. **b**, Gate R1 contains iRBCs stained with the nucleic acid dye Syto 61. The 3 dot plots to the right represent R1-gated iRBC populations reacted with no plasma (**c**), plasma from malaria-naïve American adults (**d**), and plasma from malaria-exposed Malian adults (**e**). IgG and IgM reactivities are shown in the FL1 and FL2 channels, respectively. Corresponding IgG reactivities are shown in the adjacent histograms (**f**, **g**, **h**).
(TIF)

Figure S2 **Assay-to-assay variation in % recognition values in both American and Malian adult plasma samples.** The proportion of iRBCs recognized by IgG (“% recognition”) was measured by flow cytometry. Sera from 4 malaria-naïve American adults and plasma from 4 malaria-exposed Malian adults were tested against iRBCs infected with Cambodian *P. falciparum* isolates CP803 and CP806. Each dot represents a % recognition value measured on a separate day and the bar represents the mean.
(TIF)

Figure S3 **Differences in % recognition values between paired plasma samples obtained before and after the 2009 transmission season.** Paired plasma samples from Malian children were obtained before and after the 2009 transmission season and tested against 7 parasite strains. The proportion of iRBCs recognized by IgG (“% recognition”) was measured by flow cytometry. Tukey plots show the median difference in % recognition (calculated by subtracting “pre” from “post” values) for all 176 children. The dashed line represents 0 on the y-axis. **p<0.01, ***p<0.001 via Wilcoxon match -pairs signed rank test.
(TIF)
