# Supplementary figures and images for: Effects of Age, Hemoglobin Type and Parasite Strain on IgG Recognition of Plasmodium falciparum–Infected Erythrocytes in Malian Children
Source: PLoS One. 2013 Oct 4;8(10):e76734. doi: 10.1371/journal.pone.0076734 (PMC3790723; doi:10.1371/journal.pone.0076734)

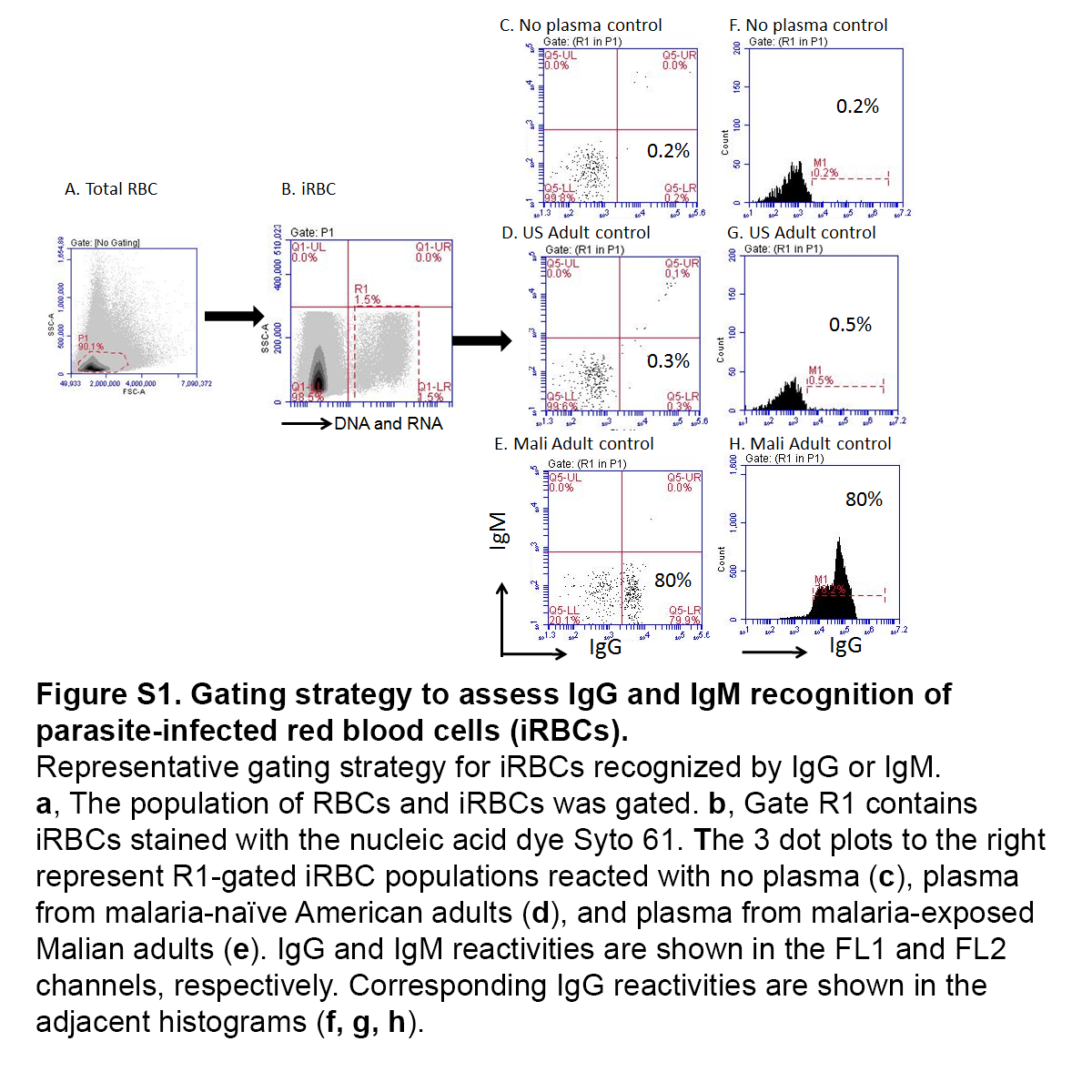

Supplement: Figure S1 — Gating strategy to assess IgG and IgM recognition of parasite-infected red blood cells (iRBCs). Representative gating strategy for iRBCs recognized by IgG or IgM. a, The population of RBCs and iRBCs was gated. b, Gate R1 contains iRBCs stained with the nucleic acid dye Syto 61. The 3 dot plots to the right represent R1-gated iRBC populations reacted with no plasma (c), plasma from malaria-naïve American adults (d), and plasma from malaria-exposed Malian adults (e). IgG and IgM reactivities are shown in the FL1 and FL2 channels, respectively. Corresponding IgG reactivities are shown in the adjacent histograms (f, g, h). (TIF) [file pone.0076734.s001.tif]

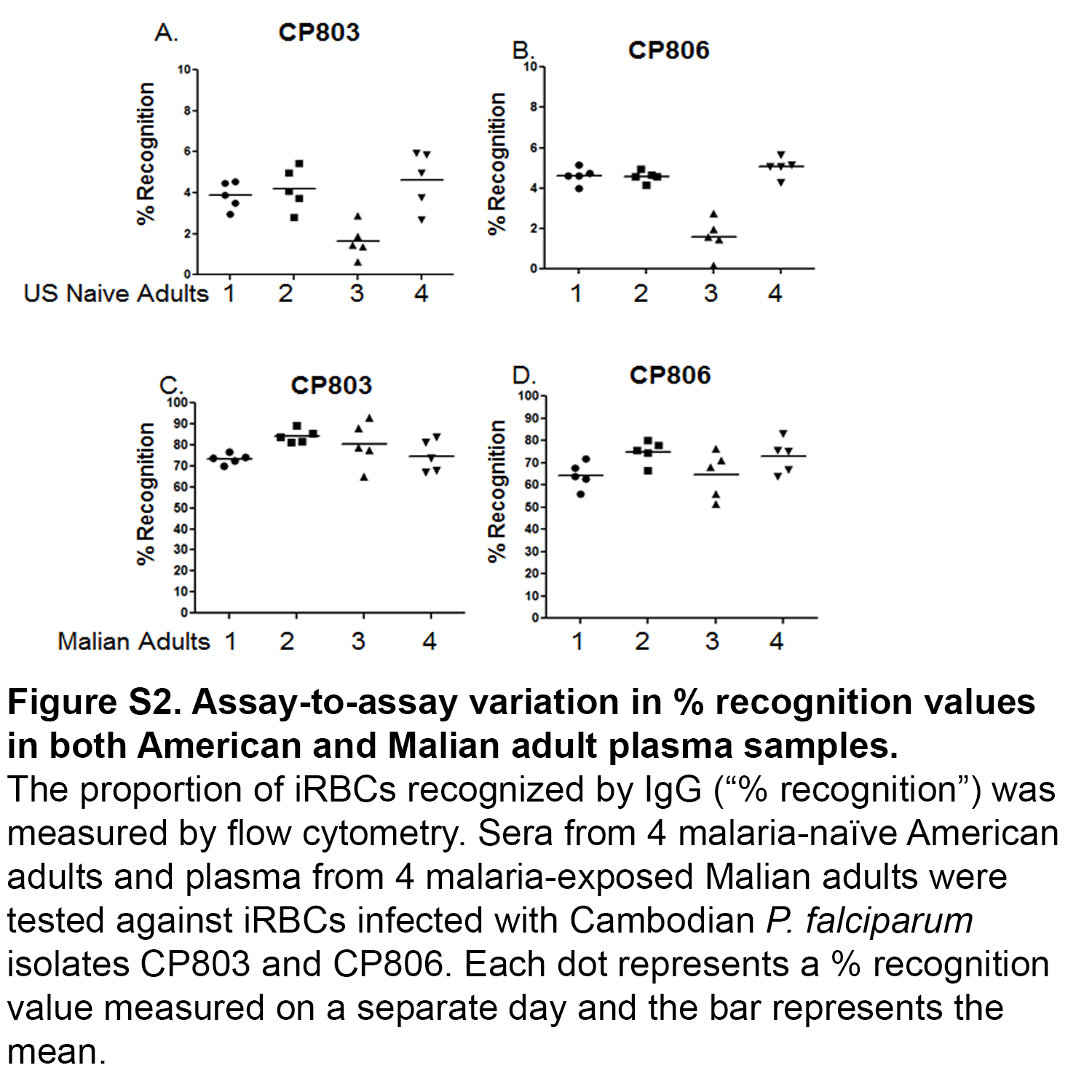

Supplement: Figure S2 — Assay-to-assay variation in % recognition values in both American and Malian adult plasma samples. The proportion of iRBCs recognized by IgG (“% recognition”) was measured by flow cytometry. Sera from 4 malaria-naïve American adults and plasma from 4 malaria-exposed Malian adults were tested against iRBCs infected with Cambodian P. falciparum isolates CP803 and CP806. Each dot represents a % recognition value measured on a separate day and the bar represents the mean. (TIF) [file pone.0076734.s002.tif]

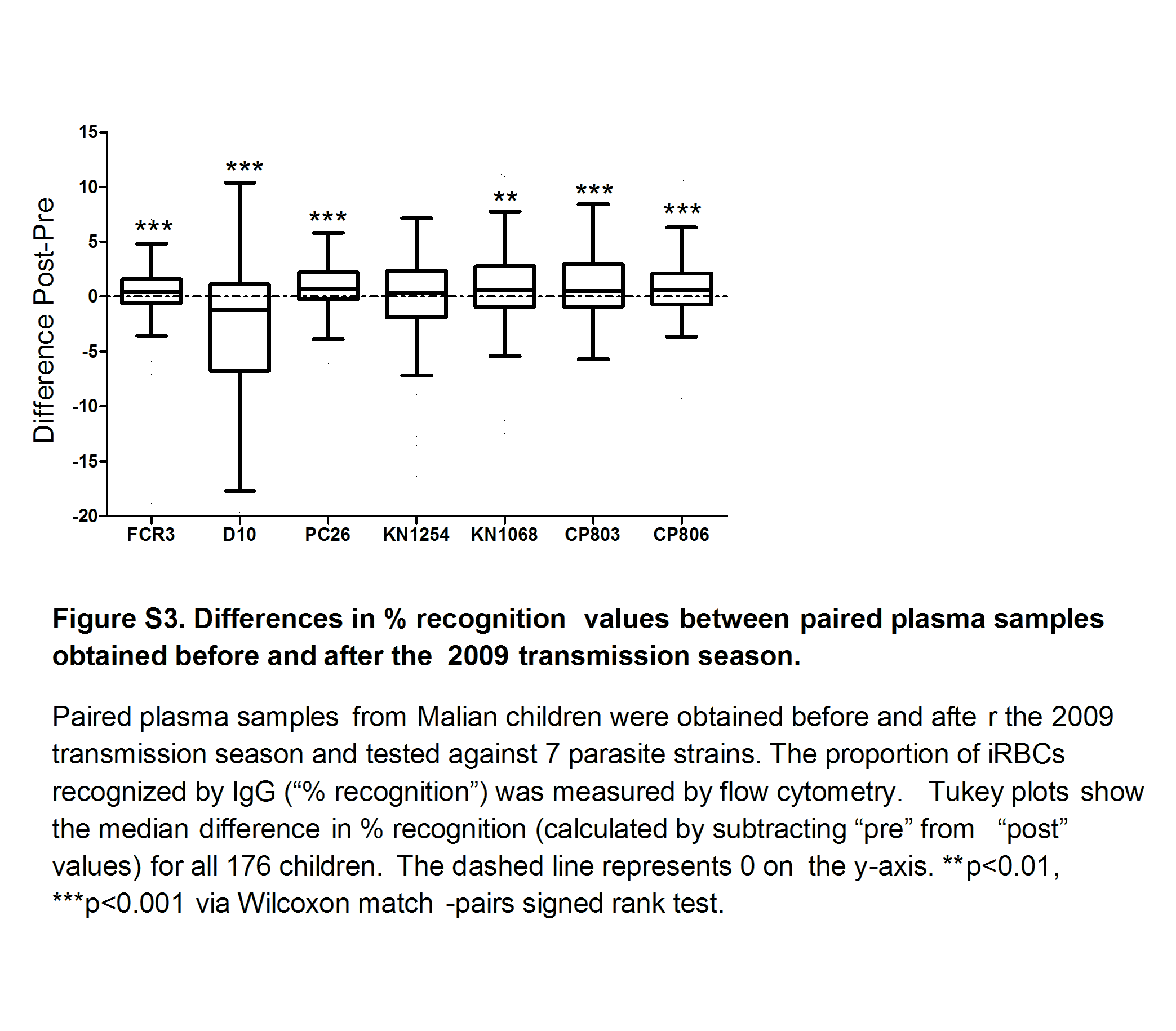

Supplement: Figure S3 — Differences in % recognition values between paired plasma samples obtained before and after the 2009 transmission season. Paired plasma samples from Malian children were obtained before and after the 2009 transmission season and tested against 7 parasite strains. The proportion of iRBCs recognized by IgG (“% recognition”) was measured by flow cytometry. Tukey plots show the median difference in % recognition (calculated by subtracting “pre” from “post” values) for all 176 children. The dashed line represents 0 on the y-axis. **p<0.01, ***p<0.001 via Wilcoxon match -pairs signed rank test. (TIF) [file pone.0076734.s003.tif]
